# Comparative Analysis of Mustardé, Fascioperichondrial Flap, and Combined Otoplasty Techniques: Clinical Outcomes and Recurrence Rates in 365 Patients

**DOI:** 10.3390/healthcare13111325

**Published:** 2025-06-03

**Authors:** Ahmet Hamdi Kepekçi

**Affiliations:** 1 Department of Audiometry, Vocational School of Health Services, Istanbul Yeni Yuzyil University, Istanbul 34010, Turkey; ahmethamdi.kepekci@yeniyuzyil.edu.tr or dr.kepekci@gmail.com; Tel.: +90-5332040462; 2 ENT Clinic, Istanbul Meltem Hospital, Istanbul 34160, Turkey

**Keywords:** otoplasty, prominent ear, Mustardé technique, fascioperichondrial flap, recurrence, surgical complications

## Abstract

Background/Objectives: Prominent ear deformity is one of the most common congenital anomalies and may lead to substantial aesthetic and psychosocial distress, particularly in children and young adults. This study evaluated the clinical outcomes of three frequently used otoplasty techniques, Mustardé sutures, fascioperichondrial flap, and their combination, concerning recurrence rates, complications, and patient satisfaction. Methods: This retrospective analysis included data from 365 patients who underwent otoplasty between 2017 and 2023 at a single institution, with all procedures performed by the same surgeon. Patients were categorized into three groups based on the surgical technique employed: Mustardé (*n* = 71), fascioperichondrial flap (*n* = 232), and a combined technique (*n* = 62). The primary outcome was the recurrence rate, while secondary outcomes included the type of suture material, complication rates, and aesthetic satisfaction. Results: The lowest recurrence rate was observed in the fascioperichondrial flap group (6.5%), followed by the combined group (8.5%) and the Mustardé group (29.6%). Logistic regression analysis revealed that the flap technique was associated with a 3.79-fold reduction in recurrence risk compared with the Mustardé method (*p* = 0.033). Overall complication rates were low across all groups. Hematoma occurred only in the flap group (1.3%), while suture extrusion and granuloma formation were exclusive to the Mustardé group. The highest aesthetic satisfaction was reported in the combined technique group. Conclusion: The fascioperichondrial flap method demonstrated statistically superior outcomes in minimizing recurrence and complications in prominent ear surgery. While the Mustardé technique demands careful patient selection due to its higher recurrence risk, the combined approach appears particularly beneficial for complex auricular deformities. The robust sample size and surgical standardization in this study provide valuable insights for surgical decision making in otoplasty. To our knowledge, this is one of the largest single-surgeon case series evaluating recurrence and complication outcomes in otoplasty.

## 1. Introduction

Prominent ear deformity is one of the most common congenital anomalies of the auricle, with an estimated prevalence of approximately 5% in the general population [1,2].

Although it does not lead to hearing impairment, its conspicuous appearance can cause significant psychosocial distress, particularly in school-aged individuals [3].

Accordingly, otoplasty is considered not merely a cosmetic procedure but also a surgical intervention that enhances the individual’s psychological well-being and social integration [4,5].

Over the past century, more than 200 surgical techniques have been described for prominent ear correction. These techniques are generally classified into two main categories: cartilage-cutting and cartilage-sparing approaches [6].

Cartilage-cutting techniques, such as those described by Chongchet and Stenström, are effective in reshaping the auricle, but they have been associated with complications, including hematoma, skin necrosis, prominent contour irregularities, and high recurrence rates [7,8].

Conversely, cartilage-sparing techniques based on suture molding, such as the Mustardé and Furnas methods, offer the advantages of preserving tissue integrity while providing a more natural antihelical fold and causing less trauma to the cartilage [9,10,11].

Nonetheless, these techniques are not without drawbacks. Reported complications include suture extrusion, granuloma formation, and recurrence rates of up to 25% [12,13,14].

To address these limitations, several supportive techniques have been introduced, with particular emphasis on the postauricular fascial flap and its variations (e.g., perichondrio-adipo-dermal flap and modified bilateral fascioperichondrial flap). These methods aim to reduce both recurrence and suture-related complications by providing a well-vascularized tissue barrier over the suture line [15,16,17,18,19].

Despite these advances, there is still no clear consensus on the most effective surgical approach for minimizing recurrence and complications. While some authors favor the classic Mustardé method for its simplicity and short learning curve [20], others advocate for fascial flap-assisted techniques due to their lower complication and recurrence rates [21,22].

Moreover, surgical success depends not only on the technique employed but also on a range of patient- and procedure-specific factors, including patient age, cartilage rigidity, suture material, surgeon experience, and adequate preoperative evaluation of lobular prominence [23,24].

This study hypothesized that the fascioperichondrial flap technique significantly reduces the recurrence rate compared with the Mustardé method. To test this hypothesis, we retrospectively compared the clinical outcomes of three commonly used otoplasty techniques: Mustardé, fascioperichondrial flap, and a combined method in patients with prominent ear deformity. The effects of each technique on recurrence, complications, and aesthetic satisfaction were assessed.

## 2. Materials and Methods

This retrospective study analyzed the clinical data of 365 patients who underwent otoplasty for prominent ear deformity between 2017 and 2023. All procedures were performed at a single center by the same surgeon. Ethical approval was obtained from the institutional review board, and written informed consent was collected from all participants.

### 2.1. Patient Selection

**Inclusion Criteria:** Patients aged between 6 and 60 years with either unilateral or bilateral prominent ear deformity who had undergone primary otoplasty and had a minimum postoperative follow-up of six months were included.

**Exclusion Criteria:** Patients were excluded if they had previously undergone revision (secondary) otoplasty, presented with syndromic auricular anomalies (e.g., microtia or anotia), had less than six months of postoperative follow-up, or underwent cartilage resection during surgery.

### 2.2. Surgical Techniques

Patients were categorized into three groups based on the surgical approach: the Mustardé group, the fascioperichondrial flap group, and the combined technique group. All procedures were performed under sterile conditions via a postauricular approach, using either local or general anesthesia. A single experienced surgeon carried out all operations to ensure technical consistency.

(a) **Mustardé Technique**

The Mustardé technique involves reshaping the auricle by placing horizontal mattress sutures on the posterior surface of the cartilage without incising the anterior aspect. After a postauricular skin incision and dissection down to the conchal cartilage, 3 to 5 mattress sutures were placed along the medial and lateral crura to reconstruct the antihelical fold.

Non-absorbable polypropylene sutures (typically 4–0 or 5–0) were used. No scoring, rasping, or resection of the cartilage was performed. After achieving hemostasis, the incision was closed with non-absorbable monofilament sutures (3–0 Propilen, Doğsan Tıbbi Malzeme Sanayi A.Ş., Trabzon, Turkey) without the use of drains.

Although this technique effectively repositions the auricle closer to the mastoid region, direct placement of sutures beneath the skin may increase the risk of long-term complications, such as suture extrusion and recurrence [12,14].

(b) **Fascioperichondrial Flap Technique**

This method is based on reinforcing the suture line with a vascularized fascioperichondrial flap raised from the postauricular region.

A “fish-mouth”-shaped elliptical incision was made, and the overlying skin was excised with a scalpel. The underlying fascioperichondrial layer was then elevated in a single dissection plane as a single-pedicle, inverted “U”-shaped flap.

This flap was designed to provide both mechanical stability and a biological barrier between the suture line and skin, thereby reducing the likelihood of suture-related complications.

**Figure 1:**
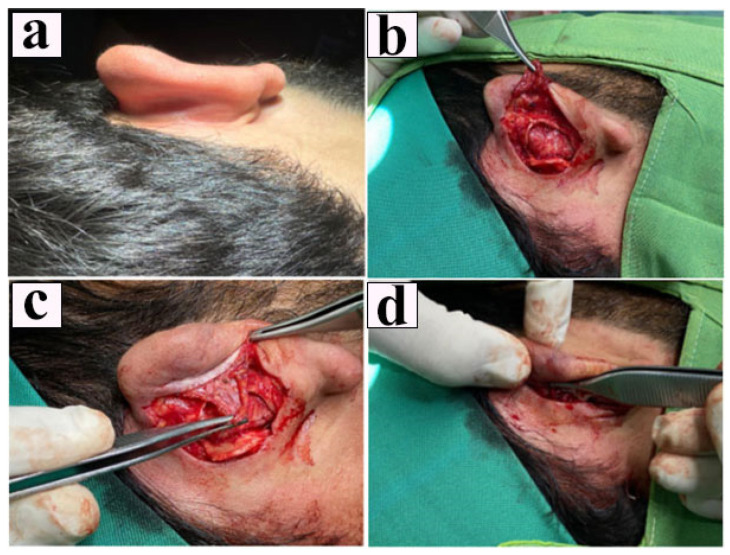
Sequential stages of fascioperichondrial flap application during otoplasty. (**a**) Preoperative appearance demonstrating the lateral protrusion characteristic of prominent ear deformity in the right auricle. (**b**) Elevation and dissection of the fascioperichondrial plane following a posterior skin incision. (**c**) Intraoperative view of the prepared deep plane intended for antihelical fold reconstruction. (**d**) Rotation of the fascioperichondrial flap over the suture line, followed by final assessment before skin closure.

No drains were placed. The incision was closed with non-absorbable monofilament sutures, and a compressive dressing was applied postoperatively (Figure 1).

(c) **Combined Technique (Mustardé + Fascioperichondrial Flap)**

In this group, the Mustardé suturing technique and fascioperichondrial flap application were performed simultaneously. This combined approach was particularly preferred in patients with conchal hypertrophy or marked conchomastoid angle deformities. The procedure began with a postauricular incision, followed by a dissection of the skin and subcutaneous tissue to expose the conchal region.

In cases with a significant conchal bowl width, conchomastoid sutures were placed between the concha and the mastoid to anchor the auricle toward the mastoid surface. Subsequently, horizontal mattress sutures were placed in accordance with the Mustardé technique to reconstruct the antihelical fold. In the same surgical session, a fascioperichondrial flap, prepared as previously described, was draped over the suture line to provide reinforcement. This approach allowed for the simultaneous narrowing of the conchal angle and restoration of the antihelical contour.

By interposing the flap, direct contact between the sutures and the skin was avoided, thereby aiming to reduce suture-related complications. Upon completion of the procedure, the incision was closed using monofilament sutures, and a compressive bandage was applied during the postoperative period.

According to the literature, combined techniques have been shown to reduce recurrence rates compared with the Mustardé method alone, although they may not be as effective as the fascioperichondrial flap technique [18,19]. Therefore, the combined technique is considered a suitable option in cases involving complex morphological deformities.

### 2.3. Evaluation Criteria

The data for each patient were recorded using eight primary parameters: age, gender, the surgical technique applied (Mustardé, fascioperichondrial flap, or the combined method), the type of suture material used (e.g., polydioxanone or propylene), the duration of postoperative follow-up, the occurrence of surgical complications (hematoma, suture extrusion, abnormal scar formation, and suture-line granuloma), the presence of recurrence (defined as loss of the achieved aesthetic correction), and the level of aesthetic satisfaction.

Recurrence was defined as a reopening of the antihelical fold or anterior rotation of the auricle within the first six months following surgery. Aesthetic dissatisfaction was assessed based on patient-reported dissatisfaction with auricular symmetry or overall appearance.

In pediatric patients under the age of 12, aesthetic satisfaction was assessed indirectly through proxy reporting by parents or legal guardians, based on observations of postoperative ear symmetry, positioning, and the child’s behavior (e.g., whether the child willingly showed their ears or expressed satisfaction or complaints).

### 2.4. Postoperative Care

All patients followed a standardized postoperative protocol regardless of the surgical technique applied. The care process was divided into phases as follows.

Immediate Phase (Days 0–3):

A custom-made compressive elastic bandage was applied immediately after surgery and left in place continuously for the first 72 h. The clinical team prescribed oral antibiotics (usually Augmentin, GlaxoSmithKline İlaçları San. ve Tic. A.Ş., Istanbul, Turkey) for 3–5 days to prevent postoperative infections.

Early Follow-Up (Days 4–11):

The surgical team removed the initial bandage under sterile conditions, examined the wound site, and applied sterile gauze when necessary. When needed, sterile gauze was applied. The team applied a topical antibiotic ointment (e.g., Fucicort, LEO Pharma İlaç Tic. Ltd. Şti., Istanbul, Turkey) to the incision line for 5–7 days and managed pain with oral analgesics, such as Parol (paracetamol, Atabay İlaç Fabrikası A.Ş., Istanbul, Turkey) or Dolven (ibuprofen, Sanofi Aventis İlaçları Ltd. Şti., Istanbul, Turkey).

Late Compression Phase (Days 12–90):

From postoperative day 12 onward, the clinical team recommended using a compression bandage only at night, continuing this regimen through day 90. The bandage was selected to maintain the ear’s shape without causing pressure or discomfort.

Follow-Up Visits and Monitoring:

The surgical team routinely evaluated patients on postoperative day 4, at the 1-month mark, and again at 3 months to monitor healing and detect any complications. During each follow-up visit, the surgical team actively monitored common complications, such as hematoma, infection, suture extrusion, asymmetry, and recurrence.

General Precautions:

The surgical team advised patients to avoid contact sports and strenuous activities for at least 3 weeks after the operation to prevent trauma to the surgical site. Additionally, protective measures, such as over-the-ear silicone shields, were recommended during showers until day 11 to prevent wound contamination.

### 2.5. Statistical Analysis

All statistical analyses were conducted using IBM SPSS Statistics Version 25.0 (IBM Corp., Armonk, NY, USA). We used descriptive statistics—the mean, standard deviation, frequency, and percentage—to summarize the data. The chi-square and Fisher’s exact tests examined relationships between categorical variables. To assess the effect of surgical techniques on recurrence, we performed binary logistic regression, as recurrence was defined as a dichotomous outcome (1 = recurrence; 0 = no recurrence). Odds ratios (ORs) and 95% confidence intervals were calculated to quantify the associations between variables. An OR above 1 indicated increased odds, while an OR below 1 indicated reduced odds of recurrence [25].

Additionally, we used linear regression to evaluate whether patient age, treated as a continuous variable, was associated with recurrence. A *p*-value of <0.05 was considered statistically significant for all analyses.

All patient data were anonymized per the Declaration of Helsinki; ethical standards were followed throughout, and written informed consent was obtained from all participants.

## 3. Results

### 3.1. Demographic Characteristics

A total of 365 patients were included in this study. Of these, 62.5% were male (*n* = 228) and 37.5% were female (*n* = 137). The age distribution was as follows: 11.0% were aged 6–18 years, 38.1% were aged 19–25 years, 36.4% were aged 26–35 years, and 14.5% were aged 36–60 years (Table 1).

### 3.2. Surgical Techniques and Overall Outcomes

The fascioperichondrial flap technique was applied in 63.6% of cases, the Mustardé technique in 19.5%, and the combined technique in 17.0%. The overall recurrence rate was 16.2% (*n* = 59), the asymmetry rate was 6.0% (*n* = 22), and the rate of aesthetically unsatisfactory outcomes was 9.6% (*n* = 35) (Table 2).

### 3.3. Effect of Gender and Age on Recurrence

Gender had no statistically significant effect on recurrence (*p* = 0.560). Recurrence was observed in 66.1% of males and 33.9% of females. Logistic regression analysis also found no significant association between age and recurrence (*p* = 0.603) (Table 3 and Table 4).

### 3.4. Complications

Complications were rare in all groups. Hematoma occurred only in the flap group (1.3%). Suture extrusion, abnormal scarring, and suture line granuloma were observed in a few cases. Asymmetry was most frequently seen in the Mustardé group (8.5%), followed by the flap group (6.5%) and the combined group (1.6%). Unsatisfactory aesthetic outcomes were most frequently reported in the combined group (12.9%).

### 3.5. Effect of Surgical Technique on Recurrence

Logistic regression analysis revealed that the recurrence rate in the fascioperichondrial flap group was 3.79 times lower than in the Mustardé group (*p* = 0.033; OR = 3.796; 95% CI: 1.996–7.222). There was no statistically significant difference between the combined technique and Mustardé (*p* = 0.296) (Table 5).

### 3.6. Hypothesis Testing

The hypothesis that “the Mustardé technique results in higher recurrence than the fascioperichondrial flap” was accepted as statistically significant. However, since the difference between the Mustardé and combined techniques was not significant, the second hypothesis was rejected (Table 6).

## 4. Discussion

To date, more than 200 techniques have been described for the surgical correction of prominent ear deformity. This wide variety indicates an ongoing lack of consensus regarding the ideal surgical approach [1]. Currently, the most commonly used methods include Mustardé, Furnas, fascioperichondrial flap, and their combinations [6,9,18].

In this study, Mustardé, fascioperichondrial flap, and the combined techniques were compared in a large series of 365 cases, revealing significant differences in recurrence, complications, and aesthetic satisfaction. The lowest recurrence rate was observed in the fascioperichondrial flap group (6.5%). According to logistic regression analysis, this technique reduced the risk of recurrence by 3.79 times compared with the Mustardé method. This suggests that supporting the suture line with a vascularized soft tissue layer enhances tissue stability, helps distribute mechanical stress, and suppresses the local inflammatory response, thereby minimizing granulation tissue formation around the sutures. Histologically, the vascularized fascia placed in this area functions as a non-epithelial buffer that separates the suture material from the overlying skin, thereby reducing the risk of extrusion and recurrence [15].

The recurrence rate in the Mustardé group was 29.6%, consistent with the 25% level reported in the literature [12,13,21]. The success of the Mustardé technique is known to be influenced by several variables, including the surgeon’s experience, patient age, cartilage elasticity, and the suture material [7,10,22]. In adult patients with rigid cartilage, the reshaping achieved through sutures tends to be less permanent, increasing the risk of recurrence [23].

Suture selection also plays a critical role in surgical outcomes. Cagici et al. [26] demonstrated that long-term absorbable sutures such as polydioxanone (PDS) induce less inflammatory reaction and promote better tissue adaptation compared with non-absorbable materials like polypropylene (propylene). We observed a lower recurrence rate and fewer cases of suture extrusion in patients who received PDS sutures. Without objective photographic validation or standardized aesthetic scoring tools, the excessive reliance on patient-reported satisfaction may have introduced subjective bias to the outcome assessment.

In the combined technique group, the recurrence rate was 8.5%, which, although lower than that of Mustardé alone, did not reach the level achieved with the fascioperichondrial flap. Although this difference was not statistically significant, the combined technique may still be advantageous in complex cases involving conchal hypertrophy or angular deformities [18,19]. While the logistic regression analysis did not demonstrate a statistically significant difference between the combined and Mustardé techniques (*p* = 0.296), the observed recurrence rate in the combined group (8.5%) was substantially lower than in the Mustardé group (29.6%). This discrepancy highlights the potential divergence between statistical significance and clinical relevance. The numerical difference, although not reaching statistical significance, may indicate a meaningful clinical advantage of the combined approach, particularly in anatomically demanding cases such as those with severe conchal hypertrophy or absent antihelical folds. The lack of statistical significance may be due to the smaller sample size of the combined group, potentially underpowering the analysis. These findings suggest that the combined technique could offer improved outcomes in selected patients, and this hypothesis warrants further investigation in larger, prospective studies. Nevertheless, its impact on recurrence appears less pronounced than that of the flap method alone.

Complication rates were low across all groups. This is likely attributable to the fact that all procedures were performed by a single surgeon following a standardized protocol. The hematoma rate observed in our study was 1.3%, notably lower than the 3–8% range reported in the literature [27,28,29], and occurred only in the flap group. Suture extrusion and granuloma formation were observed exclusively in the Mustardé group, likely due to the absence of soft-tissue coverage.

Gender and age did not show a statistically significant effect on recurrence, consistent with the findings of Binet et al. [14]. Furthermore, previous reports have suggested that insufficient consideration of lobule prominence may adversely affect postoperative symmetry and aesthetic satisfaction [24]. In the absence of objective photographic validation or standardized aesthetic scoring tools, the exclusive reliance on patient-reported satisfaction may have introduced subjective bias in outcome assessment. In our series, the combined technique group exhibited the lowest asymmetry rate (1.6%).

### 4.1. Mechanistic Perspective

The success of the fascioperichondrial flap technique is likely attributable to its dual role: mechanically distributing tension and biologically minimizing inflammation by covering the suture line with a vascularized soft-tissue layer. This coverage promotes wound healing and prevents extrusion and granuloma formation by isolating the sutures from direct skin contact. Histologically, the area is characterized by reduced fibroblast proliferation and more balanced collagen organization, potentially improving long-term shape stability.

### 4.2. Recommendations for Future Research

The retrospective nature of this study limits causal inferences. Additionally, the assessment of aesthetic satisfaction relied on subjective patient feedback. Future research should include prospective, randomized controlled trials and histopathological evaluations of the tissue response to sutures in flap-supported versus non-supported groups.

### 4.3. Contribution to the Literature

This study presents one of the largest retrospective series (*n* = 365) comparing the Mustardé, fascioperichondrial flap, and combined otoplasty techniques in terms of recurrence, complications, and aesthetic outcomes. The standardization achieved by using a single surgeon strengthens the reliability of the data. The finding that the fascioperichondrial flap technique significantly reduces recurrence rates offers novel comparative insights and addresses a gap in the current literature. Furthermore, the inclusion of the suture material as a variable provides an updated perspective on technique selection and personalized surgical planning. Overall, this study makes an original and high-value contribution to clinical decision making in otoplasty.

### 4.4. Study Limitations

The key strengths of this study include its large sample size and procedural consistency. However, limitations must be acknowledged. As a retrospective design, this study is subject to potential observer bias, missing data, and selection bias. The mean follow-up period was limited to six months, preventing a long-term outcome evaluation such as late recurrence or suture extrusion. The uneven distribution of patients among groups, particularly the smaller sample size of the Mustardé group, may have reduced the statistical power of intergroup comparisons. Additionally, aesthetic satisfaction was assessed subjectively without objective evaluation tools. Lastly, the single-center, single-surgeon nature of this study may limit the generalizability of the findings.

This study benefited from the large sample size and consistent surgical technique performed by a single experienced surgeon, enhancing its internal validity. However, several limitations must be acknowledged.

First, retrospective design inherently carries risks of recall bias, selection bias, and incomplete documentation. Although we standardized the surgical techniques and follow-up durations to mitigate these issues, the study design prevents definitive causal inferences.

Second, the mean follow-up period was six months, which, while sufficient to evaluate early complications such as hematoma and asymmetry, may be insufficient to detect late-onset issues such as suture extrusion or granuloma formation. We used this timeframe consistently to reduce attrition bias due to limitations in the availability of long-term follow-up data. Future prospective studies with extended follow-up are necessary to assess the durability of surgical outcomes.

Third, the distribution of patients across the surgical groups was unbalanced, with fewer patients in the Mustardé and combined groups compared with the fascioperichondrial flap group. This disparity may have reduced the statistical power to detect differences, particularly in rare complications. We applied chi-square and logistic regression analyses to adjust for this imbalance; however, we advise interpreting the findings with borderline significance cautiously. Future research should employ stratified or purposive sampling designs to ensure equal group sizes and increase generalizability.

Fourth, aesthetic satisfaction was assessed solely through subjective patient feedback without using objective photographic scales or blind evaluator scoring, which may introduce bias. Future studies should use standardized aesthetic assessment tools to ensure a more reliable evaluation of outcomes.

Lastly, although we recorded the suture materials, multiple suture types were applied in some patients. This limitation prevented us from making a meaningful statistical comparison between the groups. We observed that surgeons more commonly used PDS sutures in the flap and combined groups, whereas they preferred polypropylene sutures in the Mustardé group. However, these observations should be interpreted cautiously due to the lack of formal statistical testing.

### 4.5. Clinical Implications and Recommendations

The findings demonstrate that the choice of surgical technique significantly influences not only the aesthetic outcome but also recurrence and complication rates. The fascioperichondrial flap technique offers a clear advantage over suture-based approaches, making it a strong candidate for routine use in high-risk or complex cases.

Surgical planning should be individualized based on factors such as deformity severity, patient age, cartilage firmness, and aesthetic expectations. As supported by the literature, the combination of a modified postauricular fascial flap with Mustardé sutures enhances aesthetic outcomes while reducing complications [30]. In anatomically complex deformities, the combined technique may serve as a balanced and effective solution. Furthermore, choosing suture materials with prolonged durability and low tissue reactivity, such as PDS, can contribute to improved surgical results. Personalized surgical strategies guided by these principles are key to maximizing both surgical efficacy and patient satisfaction.

## 5. Conclusions

The present study demonstrates that the fascioperichondrial flap technique provides a statistically significant advantage over the Mustardé method in minimizing recurrence and suture-related complications in prominent ear correction. Additionally, the combined approach proves to be a valuable alternative in anatomically complex deformities, particularly those involving conchal hypertrophy or antihelical deficiencies. These findings support the routine implementation of flap-assisted techniques, especially in patients presenting with high-risk morphological features, and underscore the importance of individualized surgical planning in optimizing clinical outcomes.

## Figures and Tables

**Table 1:** Demographic characteristics.

Gender	*n*	%
Female	137	37.5
Male	228	62.5
**Age Group**	***n***	**%**
6–18 years	40	11.0
19–25 years	139	38.1
26–35 years	133	36.4
36–60 years	53	14.5

**Table 2:** Surgical techniques and clinical outcomes.

Surgical Method	*n*	%
Fascioperichondrial flap	232	63.6
Mustardé	71	19.5
Combined	62	17.0
**Recurrence**	***n***	**%**
Yes	59	16.2
No	306	83.8
**Asymmetry**	***n***	**%**
Present	22	6.0
Absent	343	94.0
**Unsatisfactory aesthetic outcome**	***n***	**%**
Present	35	9.6
Absent	330	90.4

**Table 3:** Recurrence by gender.

Gender	Recurrence (+)	Recurrence (–)	% Recurrence	*p*-Value
Female	20	117	38.2%	
Male	39	189	61.8%	0.560

**Table 4:** Logistic regression analysis for age and recurrence *.

Variable	B	Std. Error	Beta	t	*p*
Constant	0.131	0.063		2.081	0.038
Age	0.001	0.002	0.027	0.521	0.603

*: Age was taken as a continuous variable, and linear regression was applied (F = 0.271; R^2^ = −0.002).

**Table 5:** Logistic regression analysis of technique’s impact on recurrence *.

Technique	B	Std. Error	*p*-Value	OR	95% CI for Odds Ratio	
					Lower	Upper
Mustardé	Ref.	—	—	—		
Flap	0.913	0.328	0.033	3.796	0.691	3.358
Combined	−0.421	0.403	0.296	1.524	1.996	7.222
Constant	−1.649	0.208	0.001	0.126		

*: Logistic regression analysis was applied; *n* = 365.

**Table 6:** Summary of hypothesis testing.

Hypothesis	Result
H1: Mustardé results in higher recurrence than fascioperichondrial flap	✓ Accepted
H2: Mustardé results in higher recurrence than the combined technique	✕ Rejected

## Data Availability

The data supporting the findings of this study are available from the corresponding author upon reasonable request. Requests should be directed to ahmethamdi.kepekci@yeniyuzyil.edu.tr.

## References

[1] Siegert R., Magritz R. (2019). Otoplasty and auricular reconstruction. Facial Plast. Surg..

[2] Naumann A. (2008). Otoplasty–techniques, characteristics and risks. GMS Curr. Top. Otorhinolaryngol. Head Neck Surg..

[3] Waite E., Jenkinson E., Kershaw S., Guest E. (2024). Psychosocial Interventions for Children and Young People With Visible Differences Resulting From Appearance-Altering Conditions, Injury, or Treatment Effects: An Updated Systematic Review. J. Pediatr. Psychol..

[4] Pawar S.S., Koch C.A., Murakami C. (2015). Treatment of prominent ears and otoplasty. JAMA Facial Plast. Surg..

[5] Hamdi Kepekçi A. (2024). Comparison of Quality-of-Life Changes in Otoplasty Patients of Y and Z Generations using the GBI. Glob. J. Otolaryngol..

[6] Ordon A., Wolfswinkel E., Shauly O., Gould D.J. (2019). Aesthetic otoplasty: Principles, techniques and an integrated approach to patient-centric outcomes. Aesthetic Plast. Surg..

[7] Mandal A., Bahia H., Ahmad T., Stewart K. (2006). Comparison of cartilage scoring and cartilage sparing otoplasty—A study of 203 cases. J. Plast. Reconstr. Aesthetic Surg..

[8] Abdelhalim M., Elshahat A. (2024). Rasping First Technique in Otoplasty. Egypt. J. Plast. Reconstr. Surg..

[9] Mustarde J.C. (1963). The correction of prominent ears using simple mattress sutures. Br. J. Plast. Surg..

[10] Furnas D.W. (1968). Correction of prominent ears by concha-mastoid sutures. Plast. Reconstr. Surg..

[11] Boroditsky M.L., Van Slyke A.C., Arneja J.S. (2020). Outcomes and complications of the mustardé otoplasty: A “good–fast–cheap” technique for the prominent ear deformity. Plast. Reconstr. Surg. Glob. Open.

[12] Smittenberg M.N., Marsman M., Veeger N.J., Moues C.M. (2018). Comparison of cartilage-scoring and cartilage-sparing otoplasty: A retrospective analysis of complications and aesthetic outcome of 1060 ears. Plast. Reconstr. Surg..

[13] Limandjaja G., Breugem C., Van Der Molen A.M., Kon M. (2009). Complications of otoplasty: A literature review. J. Plast. Reconstr. Aesthetic Surg..

[14] Binet A., El Ezzi O., Roessingh A.D.B. (2020). A retrospective analysis of complications and surgical outcome of 1380 ears: Experience review of paediatric otoplasty. Int. J. Pediatr. Otorhinolaryngol..

[15] Horlock N., Misra A., Gault D.T. (2001). The postauricular fascial flap as an adjunct to Mustardé and Furnas type otoplasty. Plast. Reconstr. Surg..

[16] Schaverien M., Al-Busaidi S., Stewart K. (2010). Long-term results of posterior suturing with postauricular fascial flap otoplasty. J. Plast. Reconstr. Aesthetic Surg..

[17] Sinha M., Richard B. (2012). Postauricular fascial flap and suture otoplasty: A prospective outcome study of 227 patients. J. Plast. Reconstr. Aesthetic Surg..

[18] Aysel A., Karatan B., Ergün U., Müderris T. (2023). Modified bilateral fasciaperichondrial flap for prominent ear correction. Braz. J. Otorhinolaryngol..

[19] Irkoren S., Kucukkaya D., Sivrioglu N., Ozkan H.S. (2014). Using bilaterally fascioperichondrial flaps with a distal and a proximal base combined with conventional otoplasty. Eur. Arch. Oto-Rhino-Laryngol..

[20] García-Purriños F., Raposo A., Guilllén A., Calero J., Giribet A., Barrios A. (2019). Otoplasty using the combined Mustardé-Furnas technique: Satisfaction and objective results. Aesthetic Surg. J..

[21] Basat S.O., Askeroğlu U., Aksan T., Alleyne B., Yazar M., Orman Ç., Üsçetin İ., Akan M. (2014). New otoplasty approach: A laterally based postauricular dermal flap as an addition to Mustarde and Furnas to prevent suture extrusion and recurrence. Aesthetic Plast. Surg..

[22] Tas S., Benlier E. (2016). A new way for antihelixplasty in prominent ear surgery: Modified postauricular fascial flap. Ann. Plast. Surg..

[23] Lee Y., Kim Y.S., Lee W.J., Rha D.K., Kim J. (2018). Proposal of a classification system for the assessment and treatment of prominent ear deformity. Aesthetic Plast. Surg..

[24] Ungarelli L.F., de Andrade C.Z.N., Marques E.G.S.C., Jorge J.L.G., Neto B.F.M., de Andrade G.A.M., Lima R.V.K.S., Nunes A.A., Farina J.A. (2016). Diagnosis and Prevalence of Prominent Lobules in Otoplasty: Analysis of 120 Patients with Prominent Ears. Aesthetic Plast. Surg..

[25] Aksakoğlu G. (2001). Research Techniques and Analysis Methods in Health.

[26] Cagici C.A., Cakmak O., Bal N., Yavuz H., Tuncer I. (2008). Effects of different suture materials on cartilage reshaping. Arch. Facial Plast. Surg..

[27] Cihandide E., Kayiran O., Aydin E.E., Uzunismail A. (2016). A new approach for the correction of prominent ear deformity: The distally based perichondrio-adipo-dermal flap technique. J. Craniofacial Surg..

[28] Soares C.M.C., Nassif F.D.J.D.M., Dranka D., Becker R.V., Hurtado J.M., Freitas R.d.S. (2023). Comparative study of the effectiveness of the surgical technique with and without preservation of the conchal cartilage in otoplasty through the measure of the cephalo-auricular and scapho-conchal angles. Braz. J. Otorhinolaryngol..

[29] Kurbonov U., Davlatov A., Janobilova S., Kurbanov Z., Mirshahi M. (2018). The use of temporoparietal fascia flap for surgical treatment of traumatic auricle defects. Plast. Reconstr. Surg. Glob. Open.

[30] Kepekçi A.H. (2025). Otoplasty for Prominent Ears: Surgical Approaches, Techniques, and My Personal Experiences. Turk. Klin. Ear Nose Throat-Spec. Top..

